# Psychological and Psychosocial Interventions for PTSD, Depression and Anxiety Among Children and Adolescents in Low- and Middle-Income Countries: A Meta-Analysis

**DOI:** 10.3389/fpsyt.2019.00933

**Published:** 2020-02-18

**Authors:** Jana R. Uppendahl, Cansu Alozkan-Sever, Pim Cuijpers, Ralph de Vries, Marit Sijbrandij

**Affiliations:** ^1^Department of Clinical, Neuro and Developmental Psychology, World Health Organization Collaborating Center for Research and Dissemination of Psychological Interventions, Amsterdam Public Health Research Institute, Vrije Universiteit, Amsterdam, Netherlands; ^2^Medical Library, Vrije Universiteit, Amsterdam, Netherlands

**Keywords:** low- and middle- income countries, children, adolescents, posttraumatic stress disorder, depression, anxiety, meta-analysis, psychological therapy

## Abstract

**Background:**

In low- and middle-income countries, rates of common mental health disorders are found to be very high among children and adolescents while individuals, particularly in these countries, face barriers to mental health care. In the recent years, randomized controlled trials (RCTs) have been conducted that implemented and tested different psychological and psychosocial treatment approaches to treat common mental disorders. This review aims to analyze psychological interventions among children and adolescents in low- and middle-income countries.

**Methods:**

RCTs carried out in low- and middle-income countries on psychological and psychosocial interventions for children and adolescents with symptoms of trauma- and stressor related disorders, depression or anxiety were identified in bibliographic databases. Databases were systematically searched until December 14, 2018. Effect sizes indicating differences between treatment and control groups at post-test were computed using a random-effects model. Outcomes were symptoms of depression, anxiety and posttraumatic stress disorder (PTSD).

**Results:**

Thirteen studies with a total of 2,626 participants aged between 5 and 18 years were included. Treatments varied between studies and number of treatment sessions ranged from 1 to 16. The pooled effect size, combining outcomes of depression, anxiety and PTSD of psychological or psychosocial intervention versus care-as-usual or a control conditions yielded a medium effect (*g* = 0.62; 95% CI: 0.27–0.98). Heterogeneity was very high (*I^2^* = 94.41; 95% *CI* = 80–91). The beneficial effect of interventions increased after excluding outliers (*g* = 0.72; 95% *CI*: 0.37–1.07), while heterogeneity remained high (*I^2^* = 86.12; 95% *CI* = 87–94).

**Conclusion:**

High quality RCTs investigating the effect of psychological and psychosocial interventions on PTSD, depression and anxiety among children and adolescents in low- and middle-income countries are scarce. Results of the available studies may suggest that psychological and psychosocial interventions might be more effective in reducing symptoms of anxiety, depression and PTSD compared to control conditions. Due to very high heterogeneity, this evidence must be considered with caution.

## Introduction

Approximately one third of the total population in low- and middle-income countries are children and adolescents aged below 18 (1). Future projections see a world population shift towards an older age structure (2). Currently many western countries already face high old age dependency ratios, while many low-income countries, are considered as regions with a high “child dependency ratio,” among them most sub-Saharan African countries and parts of Asia. This means that here there are more than 45 children per 100 working age individuals (ages 15 to 64) (2). These individuals, being the majority of the world’s children and adolescents, receive on average less than US$0.01 assistance for mental health (3). Yet, prevalence estimates for youth in low- and middle-income countries range from 8–27% for anxiety symptoms, 0–28% for depressive symptoms, and for posttraumatic stress disorder (PTSD) estimates range from 0.2% to as high as 87% in adolescents who experienced traumatic events (4). Furthermore, results from the Global Burden of Disease study 2013 reveal that for children and adolescents aged 10 to 19, depressive disorders are one of the leading causes of years lived with disability (5).

When comparing prevalence rates of children and adolescents in high income countries to those in low- and middle-income countries, often a large variability has been found which has largely been attributed to methodological limitations, such as that diagnostic categories often have been developed and tested in high income countries (6). These issues impose problems when comparing prevalence rates across societies (6–8).

A number of trials have been conducted to investigate the effect of psychological and psychosocial interventions. In high income countries, psychotherapies have been found to be effective in treating common mental disorders (9, 10). In low- and middle-income countries studies focusing on adult populations have shown that psychological therapies can reduce symptoms of PTSD, depression and anxiety (11). Considering the effectiveness of psychological and psychological interventions for children and adolescents in low- and middle-income settings, meta-analyses which evaluate the evidence of RCTs examining these interventions are still lacking.

The trials that have been conducted with children and adolescents implemented various forms of psychological and psychosocial treatments, such as ﻿school-based treatments, ﻿parent- and family-focused interventions, psychoeducational and/or supportive interventions, all of which can include components of cognitive behavioral and exposure based techniques, expressive techniques and mind–body oriented skills (12). Although recommendations advise adjusting an intervention to its target group by making cultural adaptions, these are not commonly implemented or are poorly reported (11, 13, 14).

The majority of the reviews that have previously been conducted to test which of these psychological treatments are most effective focused on children and adolescents who witnessed or experienced adverse events through war or humanitarian crisis (11, 13, 15, 16). However, these reviews did not take into account the general population of children and adolescent in low-and middle-income countries. Singla et al. (17)Klasen and Crombag (18) Yatham et al. (4) and Pedersen et al. (19) performed systematic reviews on interventions for youth in low- and middle-income countries, however, these reviews were not conducted as a meta-analysis, therefore, results were not reported with statistical evidence. Likewise, Purgato et al. (12) ﻿analyzed focused psychosocial interventions for children in low-resource humanitarian settings. This review was performed as an individual patient data meta-analysis in a limited target group. Results showed a small positive effect of focused psychosocial interventions on ﻿PTSD symptoms but not for depression and anxiety (11).

To our knowledge no recent meta-analysis is currently available that provides results of psychological and psychosocial treatments for children and adolescents in low- and middle-income countries for symptoms of common mental health disorders. This meta-analysis aims to review the effectiveness of psychological and psychosocial interventions for children and adolescents that are focused on the treatment of trauma- and stressor related disorders and depression and anxiety in low- and middle-income countries.

## Methods

### Eligibility Criteria

The present review was registered on PROSPERO under the following ID: CRD42019111558. No specific funding was available for this review (20).

#### Types of Studies

In accordance with the Preferred Reporting Items for Systematic Reviews and Meta-Analysis (PRISMA) we predefined our research question in regards to Population, Intervention, Comparison, Outcome and Study (PICOS) **Table 1** (21). We included studies that were conducted as randomized controlled trials (RCTs) or cluster randomized controlled trials (cRCTs) in a low- middle-income country as defined by the World Bank (22). Studies had to implement a psychological intervention and an active or non-active comparison group had to be included.

**Table 1:** PRISMA checklist.

Section/topic	#	Checklist item	Reported on page #
**Title**	
Title	1	Identify the report as a literature review.	1
**Abstract**	
Structured summary	2	Provide a structured summary including, as applicable: background; objectives; data sources; study eligibility criteria, participants, and interventions; study appraisal and synthesis methods; results; limitations; conclusions and implications of key findings;	1
**Introduction**	
Rationale	3	Describe the rationale for the review in the context of what is already known about your topic.	
Objectives	4	Provide an explicit statement of questions being addressed with reference to participants, interventions, comparisons, outcomes, and study design (PICOS).	2
**Methods**	
Eligibility criteria	5	Specify study characteristics (e.g., PICOS, length of follow-up) and report characteristics (e.g., years considered, language, publication status) used as criteria for eligibility, giving rationale.	2; 3
Information sources	6	Describe all information sources (e.g., databases with dates of coverage) in the search and date last searched.	3
Search	7	Present full electronic search strategy for at least one database, including any limits used, such that it could be repeated.	8-10
Study selection	8	State the process for selecting studies (i.e., screening, eligibility).	4
Risk of bias in individual studies	9	Describe methods used for assessing risk of bias of individual studies (including specification of whether this was done at the study or outcome level).	3
			
Risk of bias across studies	10	Specify any assessment of risk of bias that may affect the cumulative evidence (e.g., publication bias, selective reporting within studies).	4; 5
**Results**	
Study selection	11	Give numbers of studies screened, assessed for eligibility, and included in the review, with reasons for exclusions at each stage, ideally with a flow diagram.	4
Study characteristics	12	For each study, present characteristics for which data were extracted (e.g., study size, PICOS, follow-up period) and provide the citations.	4
Synthesis of results of individual studies	13	For all outcomes considered (benefits or harms), present, for each study: (a) summary of results and (b) relationship to other studies under review (e.g. agreements or disagreements in methods, sampling, data collection or findings).	5
**Discussion**	
Summary of evidence	14	Summarize the main findings including the strength of evidence for each main outcome; consider their relevance to key groups (e.g., healthcare providers, users, and policy makers).	5-7; 8; 10; 12
Limitations	15	Discuss limitations at study and outcome level (e.g., risk of bias), and at review-level (e.g., incomplete retrieval of identified research, reporting bias).	7-8
**Conclusion**	
Conclusions	16	Provide a general interpretation of the results in the context of other evidence, and implications for future research.	12

#### Types of Participants

We included children and adolescents below the age of 18 who met diagnostic criteria for any of the anxiety, depressive disorders and/or trauma- and stressor-related disorders (as classified by DSM-5) based on a clinical diagnostic interview or scored above a specified cut-off on a self-report instrument (23). Furthermore, children and adolescents had to be living in low- and middle-income countries defined by the World Bank.

#### Types of Outcome Measures

As outcomes we defined measures of symptoms of anxiety, depression or symptoms of PTSD measure by an interview or self-report instrument.

### Procedure

#### Identification of Studies

A review protocol was developed based on the PRISMA-statement. A comprehensive search was performed in the bibliographic databases PubMed, Embase.com, and EBSCO/PsycINFO, in collaboration with a medical librarian. Databases were searched from inception until December 14, 2018. The following terms were used as index terms or free-text words (including synonyms and closely related words):

“Developing countries,” “Low- and middle-income countries,” “Anxiety disorders,” “Depressive disorders,” “Children,” and “Adolescents.” The search was performed without restrictions on date, language or publication status. Duplicate articles were excluded. The complete search strings for all databases can be found in **Table 2**–**4**. Titles and abstracts were screened by one author based on pre-defined inclusion criteria. The full-texts were then retrieved from the selected studies and rated by two authors. Data then was extracted from the included studies.

**Table 2:** Search string PubMed session results (14 Dec 2018).

Search	Query	Items found
#5	#1 AND #2 AND #3 AND #4	928
#4	clinical trial*[tw] OR controlled trial*[tw] OR random*[tw] OR psycho-social intervention*[tiab] OR psychosocial intervention*[tiab] OR psychological intervention*[tiab] OR psychologic intervention*[tiab]	1,833,474
#3	child*[tw] OR adolescen*[tw] OR pediatric*[tw] OR paediatric*[tw] OR pube*[tw] OR juvenil*[tw] OR youngster*[tiab] OR kid[tiab] OR kids[tiab] OR prepube*[tiab] OR preadolescen*[tiab] OR young people*[tiab] OR minors[tiab] OR youth*[tiab] OR teen[tiab] OR teens[tiab] OR teenager*[tiab]	3,448,709
#2	“Anxiety Disorders”[Mesh] OR “Trauma and Stressor Related Disorders”[Mesh] OR “Depressive Disorder”[Mesh] OR “Depression”[Mesh] OR “Panic”[Mesh] OR “Mutism”[Mesh] OR anxiety[tiab] OR panic[tiab] OR phobic[tiab] OR phobia[tiab] OR stressor[tiab] OR post-traumatic[tiab] OR posttraumatic[tiab] OR traumatic stress[tiab] OR psychological trauma*[tiab] OR depress*[tiab] OR dysthymi*[tiab] OR dysthimi*[tiab] OR dysphori*[tiab] OR selective mutism*[tiab] OR elective mutism*[tiab]	654,463
#1	“Developing Countries”[Mesh] OR developing countr*[tiab] OR developing nation*[tiab] OR developing population*[tiab] OR developing econom*[tiab] OR undeveloped countr*[tiab] OR undeveloped nation*[tiab] OR “undeveloped economy”[tiab] OR “undeveloped economies”[tiab] OR least developed countr*[tiab] OR least developed nation*[tiab] OR “least developed economy”[tiab] OR “least developed economies”[tiab] OR less-developed countr*[tiab] OR less-developed nation*[tiab] OR “less-developed population”[tiab] OR “less-developed populations”[tiab] OR less-developed econom*[tiab] OR lesser developed countr*[tiab] OR lesser developed nation*[tiab] OR “lesser developed population”[tiab] OR “lesser developed populations”[tiab] OR “lesser developed economy”[tiab] OR “lesser developed economies”[tiab] OR under-developed countr*[tiab] OR under-developed nation*[tiab] OR underdeveloped countr*[tiab] OR underdeveloped nation*[tiab] OR underdeveloped population*[tiab] OR underdeveloped econom*[tiab] OR low income countr*[tiab] OR middle income countr*[tiab] OR low income nation*[tiab] OR middle income nation*[tiab] OR low income population*[tiab] OR middle income population*[tiab] OR low income econom*[tiab] OR middle income econom*[tiab] OR lower income countr*[tiab] OR lower income nation*[tiab] OR lower income population*[tiab] OR “lower income economy”[tiab] OR “lower income economies”[tiab] OR resource limited[tiab] OR low resource countr*[tiab] OR lower resource countr*[tiab] OR low resource nation*[tiab] OR low resource population*[tiab] OR “low resource economy”[tiab] OR “low resource economies”[tiab] OR underserved countr*[tiab] OR underserved nation*[tiab] OR underserved population*[tiab] OR “underserved economy”[tiab] OR “underserved economies”[tiab] OR “under-served country”[tiab] OR “under-served countries”[tiab] OR “under-served nation”[tiab] OR “under-served nations”[tiab] OR “under-served population”[tiab] OR “under-served populations”[tiab] OR “underserved economy”[tiab] OR “underserved economies”[tiab] OR “deprived country”[tiab] OR “deprived countries”[tiab] OR “deprived nation”[tiab] OR “deprived nations”[tiab] OR “deprived population”[tiab] OR “deprived populations”[tiab] OR “deprived economy”[tiab] OR “deprived economies”[tiab] OR poor countr*[tiab] OR poor nation*[tiab] OR poor population*[tiab] OR poor econom*[tiab] OR poorer countr*[tiab] OR poorer nation*[tiab] OR poorer population*[tiab] OR poorer econom*[tiab] OR lmic[tiab] OR lmics[tiab] OR lami[tiab] OR transitional countr*[tiab] OR “transitional nation”[tiab] OR “transitional nations”[tiab] OR transitional econom*[tiab] OR transition countr*[tiab] OR transition nation*[tiab] OR transition econom*[tiab] OR low resource setting*[tiab] OR lower resource setting*[tiab] OR middle resource setting*[tiab] OR Third World*[tiab] OR south east asia*[tw] OR middle east*[tw] OR Afghan*[tw] OR Angola*[tw] OR Angolese*[tw] OR Angolian*[tw] OR Armenia*[tw] OR Bangladesh*[tw] OR Benin*[tw] OR Bhutan*[tw] OR Birma*[tw] OR Burma*[tw] OR Birmese*[tw] OR Burmese*[tw] OR Boliv*[tw] OR Botswan*[tw] OR burkina Faso*[tw] OR Burundi*[tw] OR Cabo Verde*[tw] OR Cambod*[tw] OR Cameroon*[tw] OR Cape Verd*[tw] OR Central Africa*[tw] OR Chad[tw] OR Comoro*[tw] OR Congo*[tw] OR Cote d’Ivoire*[tw] OR Djibouti*[tw] OR East Africa*[tw] OR Eastern Africa*[tw] OR Egypt*[tw] OR El Salvador*[tw] OR Equatorial Guinea*[tw] OR Eritre*[tw] OR Ethiopia*[tw] OR Gabon*[tw] OR Gambia*[tw] OR Gaza*[tw] OR “Georgia (Republic)”[Mesh] OR Ghan*[tw] OR Guatemal*[tw] OR Guinea[tw] OR Haiti*[tw] OR Hondur*[tw] OR India*[tw] OR Indones*[tw] OR Ivory Coast*[tw] OR Kenya*[tw] OR Kiribati*[tw] OR Kosovo*[tw] OR Kyrgyz*[tw] OR Lao PDR*[tw] OR Laos*[tw] OR Lesotho*[tw] OR Liberia*[tw] OR Madagascar*[tw] OR Malaw*[tw] OR Mali[tw] OR Mauritan*[tw] OR Mauriti*[tw] OR Micronesi*[tw] OR Mocambiqu*[tw] OR Moldov*[tw] OR Mongolia*[tw] OR Morocc*[tw] OR Mozambiqu*[tw] OR Myanmar*[tw] OR Namibia*[tw] OR Nepal*[tw] OR Nicaragua*[tw] OR Niger*[tw] OR North Korea*[tw] OR Northern Korea*[tw] OR (Democratic[tiab] AND People*[tiab] AND Republic of Korea[tiab]) OR “Democratic People’s Republic of Korea”[Mesh] OR Pakistan*[tw] OR Papua New Guinea*[tw] OR Philippine*[tw] OR Principe[tw] OR Rhodesia*[tw] OR Rwanda*[tw] OR Samoa*[tw] OR Sao Tome*[tw] OR Senegal*[tw] OR Sierra Leone*[tw] OR Solomon Islands*[tw] OR Somalia*[tw] OR South Africa*[tw] OR South Sudan*[tw] OR Southern Africa*[tw] OR Sri Lanka*[tw] OR Sub Saharan Africa*[tw] OR Subsaharan Africa*[tw] OR Sudan*[tw] OR Swaziland*[tw] OR Syria*[tw] OR Tajikist*[tw] OR Tanzan*[tw] OR Timor*[tw] OR Togo*[tw] OR Tonga*[tw] OR Tunis*[tw] OR Ugand*[tw] OR Ukrain*[tw] OR Uzbekistan*[tw] OR Vanuatu*[tw] OR Vietnam*[tw] OR West Africa*[tw] OR West Bank*[tw] OR Western Africa*[tw] OR Yemen*[tw] OR Zaire*[tw] OR Zambia*[tw] OR Zimbabw*[tw]	937,841

**Table 3:** Search string embase.com session results (14 Dec 2018).

Search	Query	Items found
#5	#1 AND #2 AND #3 AND #4	1,088
#4	‘clinical trial’/exp OR ‘clinical trial*’:ab,ti,kw OR ‘controlled trial*’:ab,ti,kw OR random*:ab,ti,kw OR ‘psychosocial intervention*’:ab,ti,kw OR ‘psycho-social intervention*’:ab,ti,kw OR ‘psychological intervention*’:ab,ti,kw OR ‘psychologic intervention*’:ab,ti,kw	2,436,080
#3	‘child’/de OR ‘adolescent’/de OR child*:ab,ti,kw OR adolescen*:ab,ti,kw OR pediatric*:ab,ti,kw OR paediatric*:ab,ti,kw OR pube*:ab,ti,kw OR juvenil*:ab,ti,kw OR youngster*:ab,ti,kw OR kid:ab,ti,kw OR kids:ab,ti,kw OR prepube*:ab,ti,kw OR preadolescen*:ab,ti,kw OR ‘young people*’:ab,ti,kw OR minors:ab,ti,kw OR youth*:ab,ti,kw OR teen:ab,ti,kw OR teens:ab,ti,kw OR teenager*:ab,ti,kw	3,463,670
#2	‘anxiety disorder’/exp OR ‘depression’/exp OR ‘selective mutism’/exp OR anxiety:ab,ti,kw OR panic:ab,ti,kw OR phobic:ab,ti,kw OR phobia:ab,ti,kw OR stressor:ab,ti,kw OR ‘post-traumatic’:ab,ti,kw OR posttraumatic:ab,ti,kw OR ‘traumatic stress’:ab,ti,kw OR ‘psychological trauma*’:ab,ti,kw OR depress*:ab,ti,kw OR dysthymi*:ab,ti,kw OR dysthimi*:ab,ti,kw OR dysphori*:ab,ti,kw OR ‘selective mutism*’:ab,ti,kw OR ‘elective mutism*’:ab,ti,kw	986,338
#1	‘developing country’/exp OR ‘low income country’/exp OR ‘middle income country’/exp OR ‘developing countr*’:ab,ti,kw OR ‘developing nation*’:ab,ti,kw OR ‘developing population*’:ab,ti,kw OR ‘developing econom*’:ab,ti,kw OR ‘undeveloped countr*’:ab,ti,kw OR ‘undeveloped nation*’:ab,ti,kw OR ‘undeveloped econom*’:ab,ti,kw OR ‘least developed countr*’:ab,ti,kw OR ‘least developed nation*’:ab,ti,kw OR ‘least developed econom*’:ab,ti,kw OR ‘less-developed countr*’:ab,ti,kw OR ‘less-developed nation*’:ab,ti,kw OR ‘less-developed population*’:ab,ti,kw OR ‘less-developed econom*’:ab,ti,kw OR ‘lesser developed countr*’:ab,ti,kw OR ‘lesser developed nation*’:ab,ti,kw OR ‘lesser developed population*’:ab,ti,kw OR ‘lesser developed econom*’:ab,ti,kw OR ‘under-developed countr*’:ab,ti,kw OR ‘under-developed nation*’:ab,ti,kw OR ‘underdeveloped countr*’:ab,ti,kw OR ‘underdeveloped nation*’:ab,ti,kw OR ‘underdeveloped population*’:ab,ti,kw OR ‘underdeveloped econom*’:ab,ti,kw OR ‘low income countr*’:ab,ti,kw OR ‘middle income countr*’:ab,ti,kw OR ‘low income nation*’:ab,ti,kw OR ‘middle income nation*’:ab,ti,kw OR ‘low income population*’:ab,ti,kw OR ‘middle income population*’:ab,ti,kw OR ‘low income econom*’:ab,ti,kw OR ‘middle income econom*’:ab,ti,kw OR ‘lower income countr*’:ab,ti,kw OR ‘lower income nation*’:ab,ti,kw OR ‘lower income population*’:ab,ti,kw OR ‘lower income econom*’:ab,ti,kw OR ‘resource limited’:ab,ti,kw OR ‘low resource countr*’:ab,ti,kw OR ‘lower resource countr*’:ab,ti,kw OR ‘low resource nation*’:ab,ti,kw OR ‘low resource population*’:ab,ti,kw OR ‘low resource econom*’:ab,ti,kw OR ‘underserved countr*’:ab,ti,kw OR ‘underserved nation*’:ab,ti,kw OR ‘underserved population*’:ab,ti,kw OR ‘underserved econom*’:ab,ti,kw OR ‘under-served countr*’:ab,ti,kw OR ‘under-served nation*’:ab,ti,kw OR ‘under-served population*’:ab,ti,kw OR ‘underserved econom*’:ab,ti,kw OR ‘deprived countr*’:ab,ti,kw OR ‘deprived nation*’:ab,ti,kw OR ‘deprived population*’:ab,ti,kw OR ‘deprived econom*’:ab,ti,kw OR ‘poor countr*’:ab,ti,kw OR ‘poor nation*’:ab,ti,kw OR ‘poor population*’:ab,ti,kw OR ‘poor econom*’:ab,ti,kw OR ‘poorer countr*’:ab,ti,kw OR ‘poorer nation*’:ab,ti,kw OR ‘poorer population*’:ab,ti,kw OR ‘poorer econom*’:ab,ti,kw OR lmic:ab,ti,kw OR lmics:ab,ti,kw OR lami:ab,ti,kw OR ‘transitional countr*’:ab,ti,kw OR ‘transitional nation*’:ab,ti,kw OR ‘transitional econom*’:ab,ti,kw OR ‘transition countr*’:ab,ti,kw OR ‘transition nation*’:ab,ti,kw OR ‘transition econom*’:ab,ti,kw OR ‘low resource setting*’:ab,ti,kw OR ‘lower resource setting*’:ab,ti,kw OR ‘middle resource setting*’:ab,ti,kw OR ‘Third World*’:ab,ti,kw OR ‘south east asia*’:ab,ti,kw,de OR ‘middle east*’:ab,ti,kw,de OR Afghan*:ab,ti,kw,de OR Angola*:ab,ti,kw,de OR Angolese*:ab,ti,kw,de OR Angolian*:ab,ti,kw,de OR Armenia*:ab,ti,kw,de OR Bangladesh*:ab,ti,kw,de OR Benin*:ab,ti,kw,de OR Bhutan*:ab,ti,kw,de OR Birma*:ab,ti,kw,de OR Burma*:ab,ti,kw,de OR Birmese*:ab,ti,kw,de OR Burmese*:ab,ti,kw,de OR Boliv*:ab,ti,kw,de OR Botswan*:ab,ti,kw,de OR ‘Burkina Faso*’:ab,ti,kw,de OR Burundi*:ab,ti,kw,de OR ‘Cabo Verde*’:ab,ti,kw,de OR Cambod*:ab,ti,kw,de OR Cameroon*:ab,ti,kw,de OR ‘Cape Verd*’:ab,ti,kw,de OR ‘Central Africa*’:ab,ti,kw,de OR Chad:ab,ti,kw,de OR Comoro*:ab,ti,kw,de OR Congo*:ab,ti,kw,de OR ‘Cote d Ivoire*’:ab,ti,kw,de OR Djibouti*:ab,ti,kw,de OR ‘East Africa*’:ab,ti,kw,de OR ‘Eastern Africa*’:ab,ti,kw,de OR Egypt*:ab,ti,kw,de OR ‘El Salvador*’:ab,ti,kw,de OR ‘Equatorial Guinea*’:ab,ti,kw,de OR Eritre*:ab,ti,kw,de OR Ethiopia*:ab,ti,kw,de OR Gabon*:ab,ti,kw,de OR Gambia*:ab,ti,kw,de OR Gaza*:ab,ti,kw,de OR ‘Georgia (republic)’/exp OR Ghan*:ab,ti,kw,de OR Guatemal*:ab,ti,kw,de OR Guinea:ab,ti,kw,de OR Haiti*:ab,ti,kw,de OR Hondur*:ab,ti,kw,de OR India*:ab,ti,kw,de OR Indones*:ab,ti,kw,de OR ‘Ivory Coast*’:ab,ti,kw,de OR Kenya*:ab,ti,kw,de OR Kiribati*:ab,ti,kw,de OR Kosovo*:ab,ti,kw,de OR Kyrgyz*:ab,ti,kw,de OR ‘Lao PDR*’:ab,ti,kw,de OR Laos*:ab,ti,kw,de OR Lesotho*:ab,ti,kw,de OR Liberia*:ab,ti,kw,de OR Madagascar*:ab,ti,kw,de OR Malaw*:ab,ti,kw,de OR Mali:ab,ti,kw,de OR Mauritan*:ab,ti,kw,de OR Mauriti*:ab,ti,kw,de OR Micronesi*:ab,ti,kw,de OR Mocambiqu*:ab,ti,kw,de OR Moldov*:ab,ti,kw,de OR Mongolia*:ab,ti,kw,de OR Morocc*:ab,ti,kw,de OR Mozambiqu*:ab,ti,kw,de OR Myanmar*:ab,ti,kw,de OR Namibia*:ab,ti,kw,de OR Nepal*:ab,ti,kw,de OR Nicaragua*:ab,ti,kw,de OR Niger*:ab,ti,kw,de OR ‘North Korea*’:ab,ti,kw,de OR ‘Northern Korea*’:ab,ti,kw,de OR (Democratic:ab,ti,kw,de AND People*:ab,ti,kw,de AND ‘Republic of Korea’:ab,ti,kw,de) OR Pakistan*:ab,ti,kw,de OR ‘Papua New Guinea*’:ab,ti,kw,de OR Philippine*:ab,ti,kw,de OR Principe:ab,ti,kw,de OR Rhodesia*:ab,ti,kw,de OR Rwanda*:ab,ti,kw,de OR Samoa*:ab,ti,kw,de OR ‘Sao Tome*’:ab,ti,kw,de OR Senegal*:ab,ti,kw,de OR ‘Sierra Leone*’:ab,ti,kw,de OR ‘Solomon Islands*’:ab,ti,kw,de OR Somalia*:ab,ti,kw,de OR ‘South Africa*’:ab,ti,kw,de OR ‘South Sudan*’:ab,ti,kw,de OR ‘Southern Africa*’:ab,ti,kw,de OR ‘Sri Lanka*’:ab,ti,kw,de OR ‘Sub Saharan Africa*’:ab,ti,kw,de OR ‘Subsaharan Africa*’:ab,ti,kw,de OR Sudan*:ab,ti,kw,de OR Swaziland*:ab,ti,kw,de OR Syria*:ab,ti,kw,de OR Tajikist*:ab,ti,kw,de OR Tanzan*:ab,ti,kw,de OR Timor*:ab,ti,kw,de OR Togo*:ab,ti,kw,de OR Tonga*:ab,ti,kw,de OR Tunis*:ab,ti,kw,de OR Ugand*:ab,ti,kw,de OR Ukrain*:ab,ti,kw,de OR Uzbekistan*:ab,ti,kw,de OR Vanuatu*:ab,ti,kw,de OR Vietnam*:ab,ti,kw,de OR ‘West Africa*’:ab,ti,kw,de OR ‘West Bank*’:ab,ti,kw,de OR ‘Western Africa*’:ab,ti,kw,de OR Yemen*:ab,ti,kw,de OR Zaire*:ab,ti,kw,de OR Zambia*:ab,ti,kw,de OR Zimbabw*:ab,ti,kw,de	1,129,403

**Table 4:** Search string EBSCO/PsycINFO session results (14 Dec 2018).

Search	Query	Items found
S7	S1 AND S2 AND S5 AND S6	233
S6	DE “Clinical Trials” OR DE “Random Sampling” OR TI (“clinical trial*” OR “controlled trial*” OR random* OR “psycho-social intervention*” OR “psychosocial intervention*” OR “psychological intervention*” OR “psychologic intervention*”) OR AB (“clinical trial*” OR “controlled trial*” OR random* OR “psycho-social intervention*” OR “psychosocial intervention*” OR “psychological intervention*” OR “psychologic intervention*”) OR KW (“clinical trial*” OR “controlled trial*” OR random* OR “psycho-social intervention*” OR “psychosocial intervention*” OR “psychological intervention*” OR “psychologic intervention*”)	214,808
S5	S3 OR S4	1,082,321
S4	**Limiters** - Age Groups: Preschool Age (2-5 yrs), School Age (6-12 yrs), Adolescence (13-17 yrs)	616,658
S3	TI (child* OR adolescen* OR pediatric* OR paediatric* OR pube* OR juvenil* OR youngster* OR kid OR kids OR prepube* OR preadolescen* OR young people* OR minors OR youth* OR teen OR teens OR teenager*) OR AB (child* OR adolescen* OR pediatric* OR paediatric* OR pube* OR juvenil* OR youngster* OR kid OR kids OR prepube* OR preadolescen* OR young people* OR minors OR youth* OR teen OR teens OR teenager*) OR KW (child* OR adolescen* OR pediatric* OR paediatric* OR pube* OR juvenil* OR youngster* OR kid OR kids OR prepube* OR preadolescen* OR young people* OR minors OR youth* OR teen OR teens OR teenager*)	890,439
S2	DE “Anxiety Disorders” OR DE “Generalized Anxiety Disorder” OR DE “Panic Disorder” OR DE “Phobias” OR DE “Acrophobia” OR DE “Agoraphobia” OR DE “Claustrophobia” OR DE “Ophidiophobia” OR DE “School Phobia” OR DE “Social Phobia” OR DE “Post-Traumatic Stress” OR DE “Posttraumatic Stress Disorder” OR DE “Complex PTSD” OR DE “DESNOS” OR DE “Separation Anxiety” OR DE “Separation Anxiety Disorder” OR DE “Major Depression” OR DE “Anaclitic Depression” OR DE “Dysthymic Disorder” OR DE “Endogenous Depression” OR DE “Late Life Depression” OR DE “Postpartum Depression” OR DE “Reactive Depression” OR DE “Recurrent Depression” OR DE “Treatment Resistant Depression” OR DE “Atypical Depression” OR DE “Depression (Emotion)” OR DE “Panic” OR DE “Elective Mutism” OR TI (anxiety OR panic OR phobic OR phobia OR stressor OR “post-traumatic” OR posttraumatic OR “traumatic stress” OR “psychological trauma*” OR depress* OR dysthymi* OR dysthimi* OR dysphori* OR “selective mutism*” OR “elective mutism*”) OR AB (anxiety OR panic OR phobic OR phobia OR stressor OR “post-traumatic” OR posttraumatic OR “traumatic stress” OR “psychological trauma*” OR depress* OR dysthymi* OR dysthimi* OR dysphori* OR “selective mutism*” OR “elective mutism*”) OR KW (anxiety OR panic OR phobic OR phobia OR stressor OR “post-traumatic” OR posttraumatic OR “traumatic stress” OR “psychological trauma*” OR depress* OR dysthymi* OR dysthimi* OR dysphori* OR “selective mutism*” OR “elective mutism*”)	468,777
S1	DE “Developing Countries” OR TI (“developing countr*” OR “developing nation*” OR “developing population*” OR “developing econom*” OR “undeveloped countr*” OR “undeveloped nation*” OR “undeveloped economy” OR “undeveloped economies” OR “least developed countr*” OR “least developed nation*” OR “least developed economy” OR “least developed economies” OR “less-developed countr*” OR “less-developed nation*” OR “less-developed population” OR “less-developed populations” OR “less-developed econom*” OR “lesser developed countr*” OR “lesser developed nation*” OR “lesser developed population” OR “lesser developed populations” OR “lesser developed economy” OR “lesser developed economies” OR “under-developed countr*” OR “under-developed nation*” OR ““underdeveloped countr*” OR “underdeveloped nation*” OR “underdeveloped population*” OR “underdeveloped econom*” OR “low income countr*” OR “middle income countr*” OR “low income nation*” OR “middle income nation*” OR “low income population*” OR “middle income population*” OR “low income econom*” OR “middle income econom*” OR “lower income countr*” OR “lower income nation*” OR “lower income population*” OR “lower income economy” OR “lower income economies” OR “resource limited” OR “low resource countr*” OR “lower resource countr*” OR “low resource nation*” OR “low resource population*” OR “low resource economy” OR “low resource economies” OR “underserved countr*” OR “underserved nation*” OR “underserved population*” OR “underserved economy” OR “underserved economies” OR “under-served country” OR “under-served countries” OR “under-served nation” OR “under-served nations” OR “under-served population” OR “under-served populations” OR “underserved economy” OR “underserved economies” OR “deprived country” OR “deprived countries” OR “deprived nation” OR “deprived nations” OR “deprived population” OR “deprived populations” OR “deprived economy” OR “deprived economies” OR “poor countr*” OR “poor nation*” OR “poor population*” OR “poor econom*” OR “poorer countr*” OR “poorer nation*” OR “poorer population*” OR “poorer econom*” OR lmic OR lmics OR lami OR “transitional countr*” OR “transitional nation” OR “transitional nations” OR “transitional econom*” OR “transition countr*” OR “transition nation*” OR “transition econom*” OR “low resource setting*” OR “lower resource setting*” OR “middle resource setting*” OR “Third World*” OR “south east asia*” OR “middle east*” OR Afghan* OR Angola* OR Angolese* OR Angolian* OR Armenia* OR Bangladesh* OR Benin* OR Bhutan* OR Birma* OR Burma* OR Birmese* OR Burmese* OR Boliv* OR Botswan* OR “Burkina Faso*” OR Burundi* OR “Cabo Verde*” OR Cambod* OR Cameroon* OR “Cape Verd*” OR “Central Africa*” OR Chad OR Comoro* OR Congo* OR “Cote d’Ivoire*” OR Djibouti* OR “East Africa*” OR “Eastern Africa*” OR Egypt* OR “El Salvador*” OR “Equatorial Guinea*” OR Eritre* OR Ethiopia* OR Gabon* OR Gambia* OR Gaza* OR (Georgia AND Republic) OR Ghan* OR Guatemal* OR Guinea OR Haiti* OR Hondur* OR India* OR Indones* OR “Ivory Coast*” OR Kenya* OR Kiribati* OR Kosovo* OR Kyrgyz* OR “Lao PDR*” OR Laos* OR Lesotho* OR Liberia* OR Madagascar* OR Malaw* OR Mali OR Mauritan* OR Mauriti* OR Micronesi* OR Mocambiqu* OR Moldov* OR Mongolia* OR Morocc* OR Mozambiqu* OR Myanmar* OR Namibia* OR Nepal* OR Nicaragua* OR Niger* OR “North Korea*” OR “Northern Korea*” OR (Democratic AND People* AND Republic of Korea) OR Pakistan* OR “Papua New Guinea*” OR Philippine* OR Principe OR Rhodesia* OR Rwanda* OR Samoa* OR “Sao Tome*” OR Senegal* OR “Sierra Leone*” OR “Solomon Islands*” OR Somalia* OR “South Africa*” OR “South Sudan*” OR “Southern Africa*” OR “Sri Lanka*” OR “Sub Saharan Africa*” OR “Subsaharan Africa*” OR Sudan* OR Swaziland* OR Syria* OR Tajikist* OR Tanzan* OR Timor* OR Togo* OR Tonga* OR Tunis* OR Ugand* OR Ukrain* OR Uzbekistan* OR Vanuatu* OR Vietnam* OR “West Africa*” OR “West Bank*” OR “Western Africa*” OR Yemen* OR Zaire* OR Zambia* OR Zimbabw*) OR AB (“developing countr*” OR “developing nation*” OR “developing population*” OR “developing econom*” OR “undeveloped countr*” OR “undeveloped nation*” OR “undeveloped economy” OR “undeveloped economies” OR “least developed countr*” OR “least developed nation*” OR “least developed economy” OR “least developed economies” OR “less-developed countr*” OR “less-developed nation*” OR “less-developed population” OR “less-developed populations” OR “less-developed econom*” OR “lesser developed countr*” OR “lesser developed nation*” OR “lesser developed population” OR “lesser developed populations” OR “lesser developed economy” OR “lesser developed economies” OR “under-developed countr*” OR “under-developed nation*” OR ““underdeveloped countr*” OR “underdeveloped nation*” OR “underdeveloped population*” OR “underdeveloped econom*” OR “low income countr*” OR “middle income countr*” OR “low income nation*” OR “middle income nation*” OR “low income population*” OR “middle income population*” OR “low income econom*” OR “middle income econom*” OR “lower income countr*” OR “lower income nation*” OR “lower income population*” OR “lower income economy” OR “lower income economies” OR “resource limited” OR “low resource countr*” OR “lower resource countr*” OR “low resource nation*” OR “low resource population*” OR “low resource economy” OR “low resource economies” OR “underserved countr*” OR “underserved nation*” OR “underserved population*” OR “underserved economy” OR “underserved economies” OR “under-served country” OR “under-served countries” OR “under-served nation” OR “under-served nations” OR “under-served population” OR “under-served populations” OR “underserved economy” OR “underserved economies” OR “deprived country” OR “deprived countries” OR “deprived nation” OR “deprived nations” OR “deprived population” OR “deprived populations” OR “deprived economy” OR “deprived economies” OR “poor countr*” OR “poor nation*” OR “poor population*” OR “poor econom*” OR “poorer countr*” OR “poorer nation*” OR “poorer population*” OR “poorer econom*” OR lmic OR lmics OR lami OR “transitional countr*” OR “transitional nation” OR “transitional nations” OR “transitional econom*” OR “transition countr*” OR “transition nation*” OR “transition econom*” OR “low resource setting*” OR “lower resource setting*” OR “middle resource setting*” OR “Third World*” OR “south east asia*” OR “middle east*” OR Afghan* OR Angola* OR Angolese* OR Angolian* OR Armenia* OR Bangladesh* OR Benin* OR Bhutan* OR Birma* OR Burma* OR Birmese* OR Burmese* OR Boliv* OR Botswan* OR “Burkina Faso*” OR Burundi* OR “Cabo Verde*” OR Cambod* OR Cameroon* OR “Cape Verd*” OR “Central Africa*” OR Chad OR Comoro* OR Congo* OR “Cote d’Ivoire*” OR Djibouti* OR “East Africa*” OR “Eastern Africa*” OR Egypt* OR “El Salvador*” OR “Equatorial Guinea*” OR Eritre* OR Ethiopia* OR Gabon* OR Gambia* OR Gaza* OR (Georgia AND Republic) OR Ghan* OR Guatemal* OR Guinea OR Haiti* OR Hondur* OR India* OR Indones* OR “Ivory Coast*” OR Kenya* OR Kiribati* OR Kosovo* OR Kyrgyz* OR “Lao PDR*” OR Laos* OR Lesotho* OR Liberia* OR Madagascar* OR Malaw* OR Mali OR Mauritan* OR Mauriti* OR Micronesi* OR Mocambiqu* OR Moldov* OR Mongolia* OR Morocc* OR Mozambiqu* OR Myanmar* OR Namibia* OR Nepal* OR Nicaragua* OR Niger* OR “North Korea*” OR “Northern Korea*” OR (Democratic AND People* AND Republic of Korea) OR Pakistan* OR “Papua New Guinea*” OR Philippine* OR Principe OR Rhodesia* OR Rwanda* OR Samoa* OR “Sao Tome*” OR Senegal* OR “Sierra Leone*” OR “Solomon Islands*” OR Somalia* OR “South Africa*” OR “South Sudan*” OR “Southern Africa*” OR “Sri Lanka*” OR “Sub Saharan Africa*” OR “Subsaharan Africa*” OR Sudan* OR Swaziland* OR Syria* OR Tajikist* OR Tanzan* OR Timor* OR Togo* OR Tonga* OR Tunis* OR Ugand* OR Ukrain* OR Uzbekistan* OR Vanuatu* OR Vietnam* OR “West Africa*” OR “West Bank*” OR “Western Africa*” OR Yemen* OR Zaire* OR Zambia* OR Zimbabw*) OR KW (“developing countr*” OR “developing nation*” OR “developing population*” OR “developing econom*” OR “undeveloped countr*” OR “undeveloped nation*” OR “undeveloped economy” OR “undeveloped economies” OR “least developed countr*” OR “least developed nation*” OR “least developed economy” OR “least developed economies” OR “less-developed countr*” OR “less-developed nation*” OR “less-developed population” OR “less-developed populations” OR “less-developed econom*” OR “lesser developed countr*” OR “lesser developed nation*” OR “lesser developed population” OR “lesser developed populations” OR “lesser developed economy” OR “lesser developed economies” OR “under-developed countr*” OR “under-developed nation*” OR ““underdeveloped countr*” OR “underdeveloped nation*” OR “underdeveloped population*” OR “underdeveloped econom*” OR “low income countr*” OR “middle income countr*” OR “low income nation*” OR “middle income nation*” OR “low income population*” OR “middle income population*” OR “low income econom*” OR “middle income econom*” OR “lower income countr*” OR “lower income nation*” OR “lower income population*” OR “lower income economy” OR “lower income economies” OR “resource limited” OR “low resource countr*” OR “lower resource countr*” OR “low resource nation*” OR “low resource population*” OR “low resource economy” OR “low resource economies” OR “underserved countr*” OR “underserved nation*” OR “underserved population*” OR “underserved economy” OR “underserved economies” OR “under-served country” OR “under-served countries” OR “under-served nation” OR “under-served nations” OR “under-served population” OR “under-served populations” OR “underserved economy” OR “underserved economies” OR “deprived country” OR “deprived countries” OR “deprived nation” OR “deprived nations” OR “deprived population” OR “deprived populations” OR “deprived economy” OR “deprived economies” OR “poor countr*” OR “poor nation*” OR “poor population*” OR “poor econom*” OR “poorer countr*” OR “poorer nation*” OR “poorer population*” OR “poorer econom*” OR lmic OR lmics OR lami OR “transitional countr*” OR “transitional nation” OR “transitional nations” OR “transitional econom*” OR “transition countr*” OR “transition nation*” OR “transition econom*” OR “low resource setting*” OR “lower resource setting*” OR “middle resource setting*” OR “Third World*” OR “south east asia*” OR “middle east*” OR Afghan* OR Angola* OR Angolese* OR Angolian* OR Armenia* OR Bangladesh* OR Benin* OR Bhutan* OR Birma* OR Burma* OR Birmese* OR Burmese* OR Boliv* OR Botswan* OR “Burkina Faso*” OR Burundi* OR “Cabo Verde*” OR Cambod* OR Cameroon* OR “Cape Verd*” OR “Central Africa*” OR Chad OR Comoro* OR Congo* OR “Cote d’Ivoire*” OR Djibouti* OR “East Africa*” OR “Eastern Africa*” OR Egypt* OR “El Salvador*” OR “Equatorial Guinea*” OR Eritre* OR Ethiopia* OR Gabon* OR Gambia* OR Gaza* OR (Georgia AND Republic) OR Ghan* OR Guatemal* OR Guinea OR Haiti* OR Hondur* OR India* OR Indones* OR “Ivory Coast*” OR Kenya* OR Kiribati* OR Kosovo* OR Kyrgyz* OR “Lao PDR*” OR Laos* OR Lesotho* OR Liberia* OR Madagascar* OR Malaw* OR Mali OR Mauritan* OR Mauriti* OR Micronesi* OR Mocambiqu* OR Moldov* OR Mongolia* OR Morocc* OR Mozambiqu* OR Myanmar* OR Namibia* OR Nepal* OR Nicaragua* OR Niger* OR “North Korea*” OR “Northern Korea*” OR (Democratic AND People* AND Republic of Korea) OR Pakistan* OR “Papua New Guinea*” OR Philippine* OR Principe OR Rhodesia* OR Rwanda* OR Samoa* OR “Sao Tome*” OR Senegal* OR “Sierra Leone*” OR “Solomon Islands*” OR Somalia* OR “South Africa*” OR “South Sudan*” OR “Southern Africa*” OR “Sri Lanka*” OR “Sub Saharan Africa*” OR “Subsaharan Africa*” OR Sudan* OR Swaziland* OR Syria* OR Tajikist* OR Tanzan* OR Timor* OR Togo* OR Tonga* OR Tunis* OR Ugand* OR Ukrain* OR Uzbekistan* OR Vanuatu* OR Vietnam* OR “West Africa*” OR “West Bank*” OR “Western Africa*” OR Yemen* OR Zaire* OR Zambia* OR Zimbabw*)	68,307

#### Data Extraction

Data was extracted on an Excel sheet by two independent researchers. Information on study design, country, assessment instrument, blinding of assessors, type of comparison, type of therapy, treatment provider, fidelity assessment, theoretical background, duration of therapy, number of sessions and format of therapy, in- and exclusion criteria of participants, number of participants, gender, mean age, standard deviation and age range of participants, pre-treatment, post-treatment and follow-up assessment means standard deviations or mean differences and standard deviations of change scores for both intervention and control groups were recorded. Discrepancies were discussed and resolved.

#### Quality Assessment

Quality of studies was assessed by two independent reviewers. Version 2 of the Cochrane risk-of-bias tool (RoB 2) was used to perform quality assessment (24). Randomized controlled trials were assessed with RoB 2 for randomized controlled trials, and cluster randomized controlled trials were respectively assessed with RoB 2 for cluster randomized controlled trials. In both, assessments studies were evaluated on the following paradigms: Bias arising from the randomization process; bias arising from the timing of identification and recruitment of individual participants in relation to randomization; bias due to deviations from intended interventions; bias due to missing outcome data; bias in measurement of the outcome, and bias in selection of the reported result. All paradigms inherit specific questions concerning the study that are answered with either “yes,” “probably yes,” “no,” “probably no” or “no information.” These questions guide the decision to evaluate a paradigm, and therewith, the risk of bias in a study with either “low risk,” “some concerns” or “high risk.” The Cochrane tool provides an algorithm to guide the final evaluation into one of the three categories. Questions within the paradigms differ slightly between the assessment for individual randomized trials and cluster randomized trials. Finally, an overall rating of the study was made based on the aforementioned ratings of the five paradigms.

#### Data Analysis

Data analysis was performed with the software Comprehensive Meta-Analysis (CMA) version 3 (25). Data was compiled from Intention to treat samples (ITT) of the studies when available. Completer samples were used when ITT samples were not reported. Standardized mean differences (Hedges’ g) were calculated as a measure of effect size. Hedges’ g is calculated by first subtracting the posttest mean of the treatment group from the posttest mean of the control group and then dividing by the pooled standard deviations of both groups. In this study, this measure was utilized to indicate the difference between the treatment and control condition at post-test. In some studies, mean and standard deviations were not reported but mean change scores were provided. In these cases, Hedges’ g was calculated with the mean change scores and standard deviation differences of each group. If more than one measure of anxiety, depression or symptoms of PTSD was reported, these were all included in the analysis. A random effects model was used due to expected heterogeneity among studies. Heterogeneity was measured with the I^2^ statistics. We calculated 95% confidence intervals (CI) around I^2^ (26), using the non-central Chi squared-based approach within the heterogeneity module for Stata (27). To analyze whether characteristics of the studies predicted effect sizes, we performed subgroup analysis. Subgroup analysis was performed using the mixed effects model (28), in which effect sizes within subgroups are pooled according to the random effects model and the difference between subgroups according to a fixed effects model. Subgroup analyses were performed between cluster and individual randomized controlled trials, studies with different risk of bias assessment and between studies with a waitlist control condition and a non-waitlist control condition. Sensitivity analysis was computed in which potential outliers were excluded. Outliers were defined as studies of which the 95% CI of the effect size did not overlap with the 95% CI of the pooled effect size. Finally, publication bias was examined with Duval and Tweedie’s trim and fill procedure and the Egger’s test of asymmetry (29).

## Results

### Selection and Inclusion of Studies

**Figure 1:**
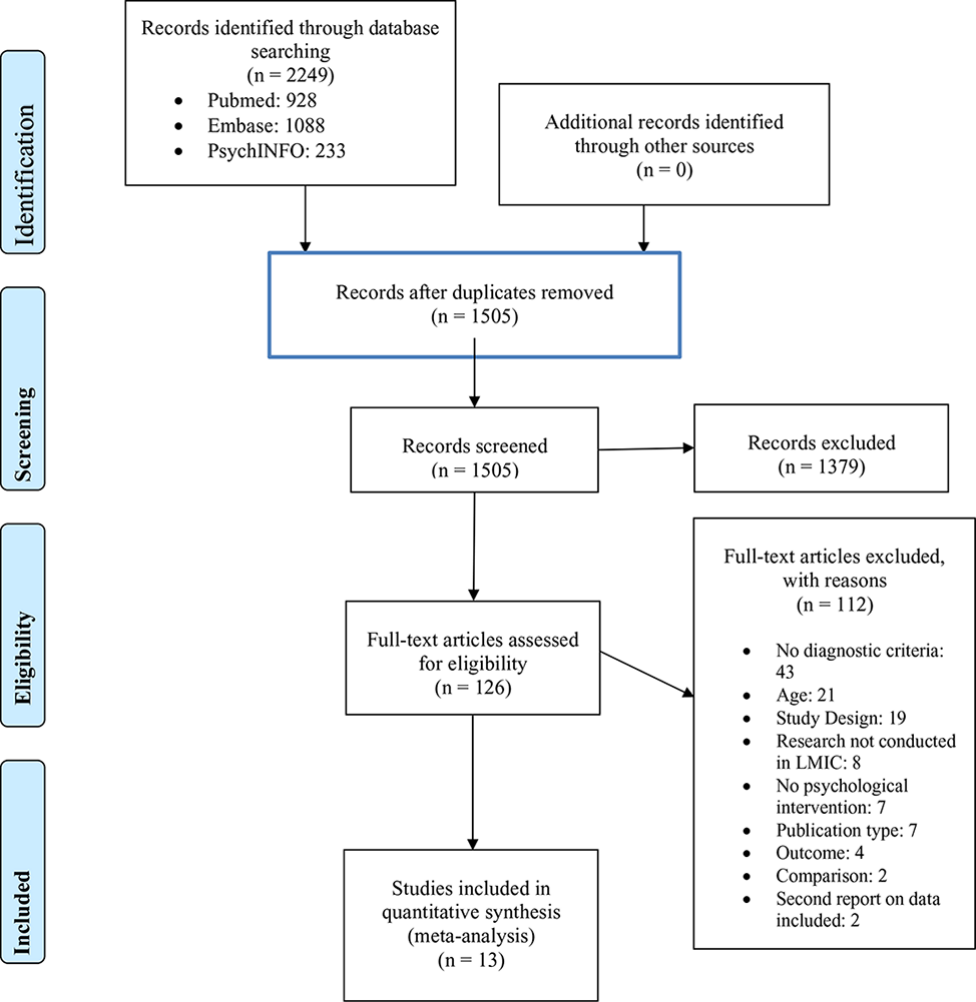
PRISMA flow diagram.

After identifying 2,249 articles in bibliographic databases, records were de-duplicated and 1,505 titles and abstracts were screened for inclusion. Thereafter, the full-text was retrieved from 126 articles and 1,379 articles were excluded. From the 126 reviewed full-texts, 13 studies were included in the meta-analysis. Reasons for excluding articles to a large extent were due to the lack of a screening assessment or criteria within the studies to ensure that individuals with symptoms of either anxiety, depression or PTSD were recruited. Other reasons to exclude articles were study design, outcome, second report on data included, research not conducted in a LMIC, no psychological treatment or publication type [See PRISMA flow diagram (21), **Figure 1**]. Seven studies were excluded based on publication type, as the way in which the study was published, e.g. as an abstract, did not provide sufficient data for the meta-analysis.

### Characteristics of the Included Studies

A total of 13 studies were included in the analysis. In all studies combined, 1,227 participants were enrolled in a psychological intervention and 1,398 participants were enrolled in a control group. One study only recruited girls. Twelve of the studies included in this review were conducted in Asia and Africa, with one study conducted in Southeastern Europe (Kosovo). Out of the studies included, seven were randomized controlled studies, and six were cluster-randomized controlled studies. The number of participants randomized in each study ranged from 15 to 221. The participants included in the studies were aged between 5 and 18 years. Outcomes for depression were reported in ten studies, anxiety in four studies and PTSD in nine studies. Solely one study reported multiple outcomes, for the outcome that was investigated (30). In that study depression was measured with three different outcomes which were all included in this meta-analysis. Psychological treatment was compared to a wait-list control condition in eight studies, four studies compared treatment to a control group and one study utilized treatment as usual (TAU) as a control group which referred to community services available in Zambia (31). The number of treatment sessions ranged from 1 to 16. One study implemented an intervention within an individual format, namely trauma focused CBT (TF-CBT), while the other studies utilized interventions within a group format. These interventions included: 1) Teaching recovery techniques (TRT), which is based on CBT with a focus on symptoms of PTSD wherein individuals are taught coping skills and relaxation techniques with the aim of gradually desensitizing individuals’ phobic avoidance behavior (32), 2) Interpersonal therapy (IPT), 3) Creative play for reducing symptoms of depression, which is facilitated through the expression of thoughts and feelings by art, music, roleplays, games, and discussions (33), 4) Mind body skills group, which also incorporates verbal and nonverbal self-expression activities with the goal to provide individuals with a coping tool to deal with experienced trauma (34), 5) Didactic therapy for the management of stress with coping and appraisal strategies, cognitive behavioral techniques and didactic presentations with work sheets on regulation of emotion and behavior (35), 6) Bibliotherapy, with the aim of reducing depressive symptoms by generating insight into ways of thinking and behaving and replacing maladaptive behavior through reading in a therapeutic setting (30), 7) Spiritual hypnosis, which included reinterpretation the meaning of the traumatic event and expressing emotions (36), 8) (37) CBT, Crisis intervention that encourages the discussion and sharing of traumatic experiences and was facilitated by the use of drawing, games, role-play and talking (38), and 9) School-based interventions which included components of CBT including exposure and creative expressive elements such as drawing and games (39–42). Characteristics of the included studies are also depicted in **Table 5**.

**Table 5:** Characteristics of included studies.

Study	Comparison	Age	Outcomes	Format	N Sessions	Country
32	Teaching recovery techniques vs WL	11–18	Depression PTSD	Group	5	Palestine
37	CBT vs WL	14–17	Depression	Group	5	Southwest Nigeria
33	IPT and creative play vs control	14–17	Depression	Group	16	Northern Uganda
34	Mind body skills group vs WL	14–18	PTSD	Group	12	Kosovo
35	Didactic therapy vs control	12–18	Depression Anxiety Stress	Group	6	Pakistan
30	Bibliotherapy vs control	13–16	Depression	Group	8	Philippines
39	School-based intervention vs WL	11–14	Anxiety Depression PTSD	Group	15	Nepal
36	Spiritual hypnosis assisted therapy vs control	6–12	Avoidance Hyperarousal Reexperiencing	Group	1	Bali
31	TF-CBT vs TAU	5–18	PTSD	Individual	NR	Zambia
38	Crisis intervention vs control	9–15	Depression PTSD	Group	7	Gaza Strip
40	School-based intervention vs WL	7–15	Anxiety Depression PTSD	Group	15	Indonesia
41	School-based intervention vs WL	9–12	Anxiety Depression PTSD	Group	15	Sri Lanka
42	School-based intervention vs WL	8–17	Depression PTSD	Group	15	Burundi
						

### Risk of Bias

**Figure 2:**
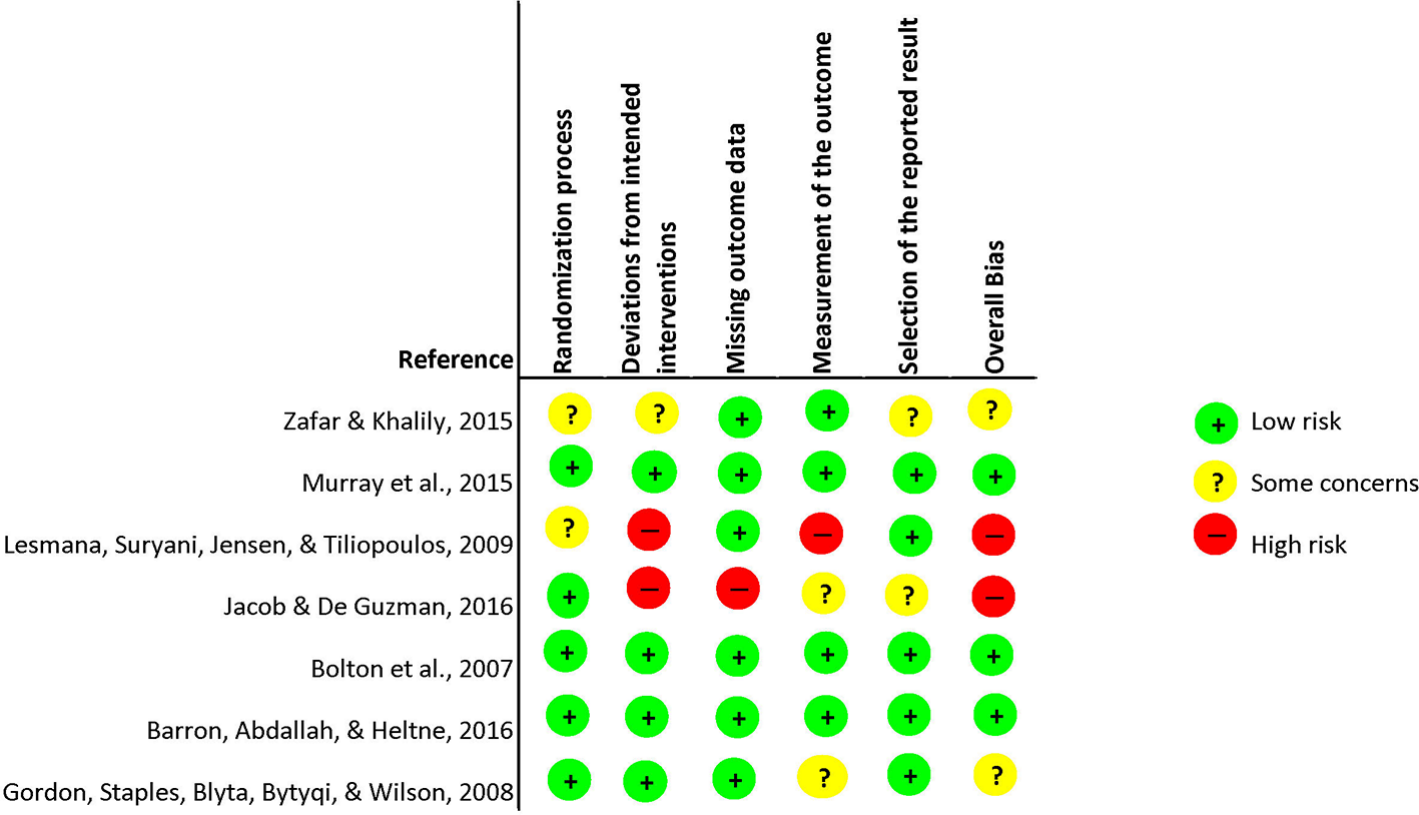
Risk of Bias Cluster Randomized Controlled Trials.

**Figure 3:**
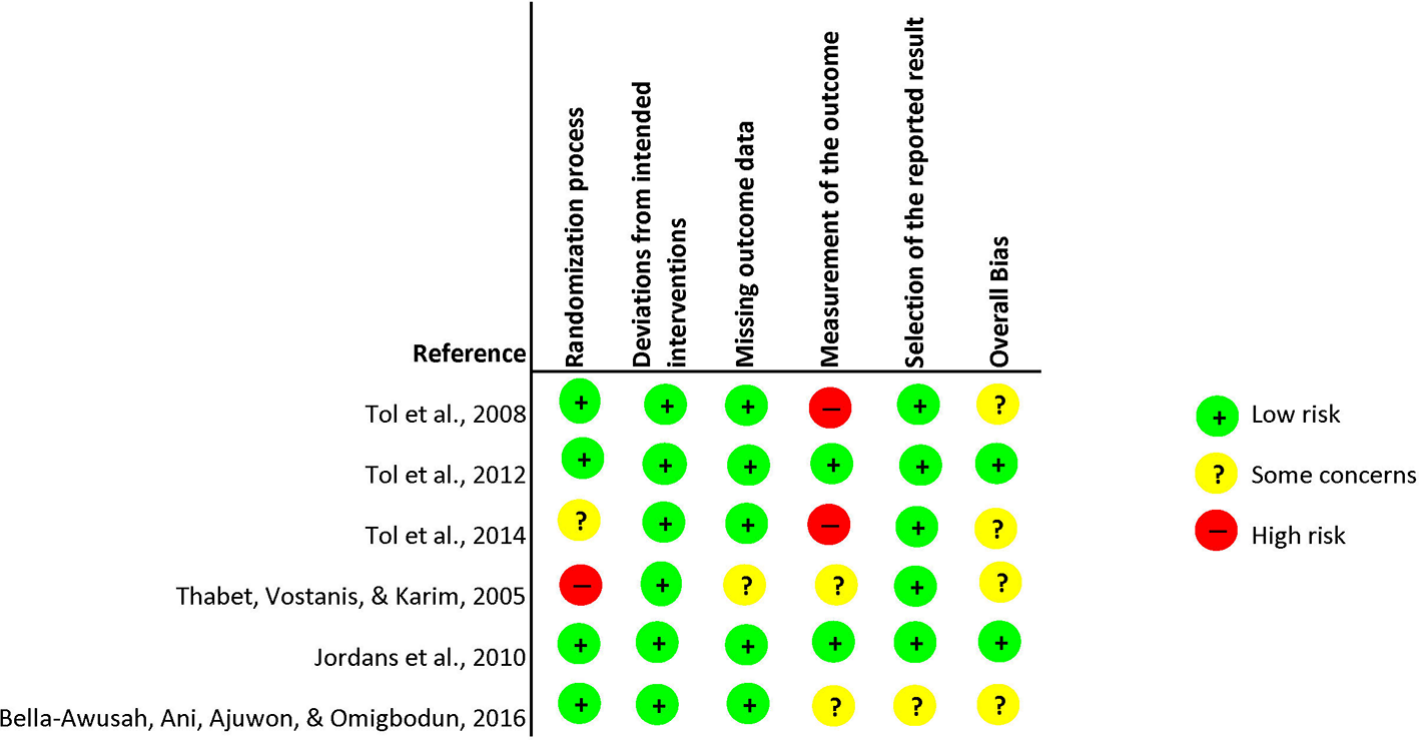
Risk of Bias Cluster Randomized Controlled Trials.

The risk of bias was assessed separately for individual and cluster randomized controlled trials. Within the individual randomized trials, two studies were rated with an overall high risk of bias. This rating was provided due to: the lack of providing clear description of the plan of analysis, the lack of indicating how many participants completed the interventions, and potential deviations from intended interventions due to awareness of treatment condition and due to a lack of information provided on the analysis used to estimate the effect of assignment to intervention. Other studies were most likely not devoid of the awareness of treatment conditions, however, combined with the aforementioned issues these studies received an overall rating of “high risk.” The major shortcomings within the randomization process and outcome measurement was that not enough information was provided to apprehend how studies randomized. In respect to the outcome measures, the major shortcoming was that assessors were not blinded. Furthermore, two studies received an overall risk of bias rating of “some concerns” due to: randomization process, deviations from intended interventions, measurement of the outcome measure and no comprehensive reporting of analysis plan and actual analysis. Three studies received an overall rating that was considered as signaling “low risk” of bias. Within the risk of bias for cluster randomized trials, four studies received an overall risk of bias rating considered as potentially incorporating “some risk.” This rating was given due to: considerations within the randomization process, no clarity whether outcome data was missing, measurement of the outcome data and potential selection of results. For graphic representation see **Figure 2** for cluster randomized- and **Figure 3** for individual randomized trials.

### Effect of Psychological and Psychosocial Interventions

**Figure 4:**
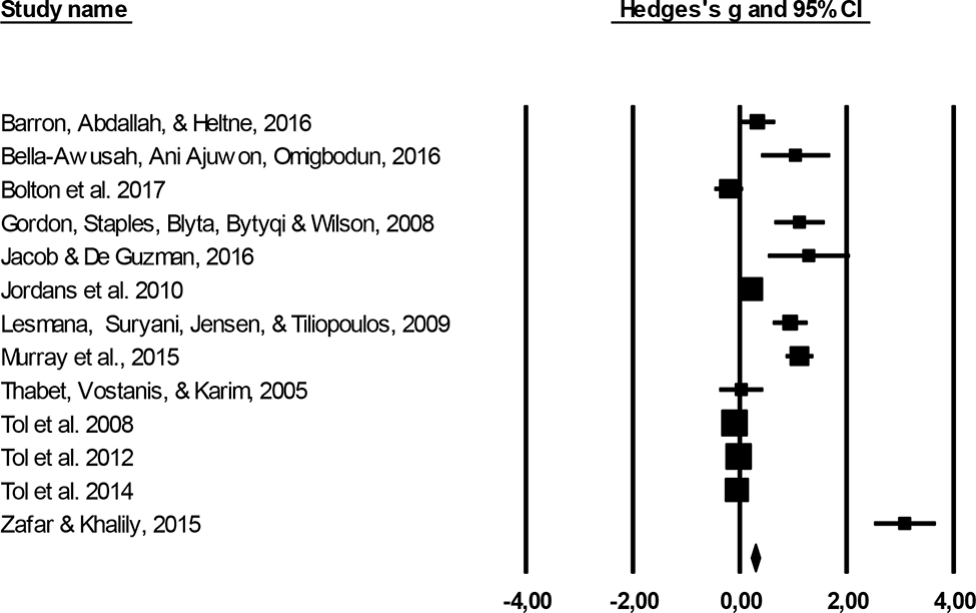
Forest plot of combined outcomes.

This effect of psychological interventions for all outcomes was (*g* = 0.62; *95% CI*: 0.27–0.98) with very high heterogeneity (*I^2^* = 94.41; *95% CI*: 80–91). After excluding outliers, the effect size increased to *g* = 0.72 (*95% CI*: 0.37–1.07), with very high heterogeneity (*I^2^* = 86.12; *95% CI*: 87–94). Five studies were defined as outliers and were excluded (See **Figure 4**).

For depression symptoms, psychological interventions yielded a medium effect size (*g* = 0.43; *95% CI*: 0.06–0.80) with high heterogeneity (*I^2^* = 94.06; *95% CI*: 85–93). Three outliers were detected and excluded, which led to a decrease in the effect size (*g* = 0.21; *95% CI*: −0.05–0.48) with a heterogeneity of *I^2^* = 80.11 (*95% CI*: 88–95).

For anxiety symptoms, psychological interventions showed a small effect for psychological treatments (*g* = 0.18; *95% CI*: 0.06–0.29) with high heterogeneity (*I^2^* = 96.46; *95% CI*: 95–98). The effect of psychological treatments for anxiety dropped to *g* = 0.06 (*95% CI*: −0.06–0.17) with low heterogeneity after excluding one outlier (*I^2^* = 0.0; *95% CI*: 0–73).

The outcomes for psychological therapies on PTSD symptoms indicated a medium effect size (*g* = 0.43; *95% CI*: 0.10–0.77) with heterogeneity (*I^2^* = 92.86; *95% CI*: 86–94). After excluding three studies, which were defined as outliers, the effect size increased to *g* = 0.50 (*95% CI*: 0.17–0.83) with high heterogeneity (*I^2^* = 85.54; *95% CI*: 91–96) (see **Table 6** and **7**).

**Table 6:** Combined outcomes of psychological and psychosocial interventions compared to control conditions.

Study	Hedges’g	CI lower limit	CI upper limit	p-value	N
32	0.33	−0.01	0.66	0.06	139
37	1.04	0.39	1.69	0.00	40
33	−0.21	−0.48	0.06	0.13	209
34	1.12	0.64	1.59	0.00	78
30	1.29	0.52	2.06	0.00	30
52	0.21	0.00	0.43	0.05	325
36	0.94	0.61	1.27	2.03	226
31	1.12	0.86	1.38	0.00	257
38	0.03	−0.38	0.44	0.89	89
40	−0.10	−0.29	0.10	0.34	403
41	−0.02	−0.22	0.18	0.85	390
42	−0.05	−0.27	0.16	0.63	329
35	3.09	2.51	3.67	0.00	100

**Table 7:** Summary of the results.

Analysis	N of studies	Hedges’g	CI	I^2^	I^2^ CI
**Combined outcomes**	13	0.62	0.27–0.98	94.41	80–91
**Combined outcomes** *Outliers excluded*	8	0.72	0.37–1.07	86.12	87–94
**Depression**	10	0.43	0.06–0.80	94.06	*85–93*
**Depression** * Outliers excluded*	7	0.21	−0.05–0.48	80.11	88–95
**Anxiety**	4	0.18	0.06–0.29	96.46	95–98
**Anxiety** *Outliers excluded*	3	0.06	−0.06–0.17	0.0	0–73
**PTSD**	9	0.43	0.10–0.77	92.86	86–94
**PTSD** * Outliers excluded*	6	0.50	0.17–0.83	85.54	91–96

Examination of the funnel plot indicated significant publication bias. Duvall and Tweedie’s trim and fill procedure suggested that four studies be imputed as a result of publication bias, which resulted in a decreased effect size of *g* = 0.16 (95% CI: −0.23 ~ 0.56). Furthermore, Egger’s test was significant with an intercept of 7.36 (95% CI: 2.49 ~ 1.87, *p* = 0.01).

### Subgroup Analysis

Effect sizes were significantly smaller when waitlist control group was used, (six studies) compared to studies using another control condition (seven studies; *p* = 0.05). Subgroup analysis also showed that individually randomized studies (seven studies) had significantly higher effect sizes than cRCTs (six studies; *p* < 0.005). Lastly, studies with high risk of bias (two studies), some risk of bias (six studies) and low risk of bias (five studies) were compared. These results indicated that quality of study was not significantly associated with effect size (*p* = 0.43).

## Discussion

This meta-analysis reviewed the effect of psychological interventions on symptoms of depression, anxiety, and PTSD in children and adolescents in low- and middle-income countries. Thirteen studies with a total of 2,626 participants fulfilled the predefined inclusion criteria and were included in the analysis. Notably, interventions within the included studies varied greatly from each other. They targeted different symptoms (e.g., anxiety, depression or PTSD) and utilized different treatment approaches with variations in session frequency. Most of the interventions were ﻿low-threshold interventions such as school-based interventions. However, some were also more specialized mental health interventions, such as TF-CBT and IPT. Due to very high heterogeneity the results of our meta-analysis should be interpreted with caution.

The result for all pooled outcomes showed a medium to large effect for psychological interventions compared to control conditions. Also, for the outcomes separately a beneficial effect of intervention could be observed. Interventions had a positive effect on the reduction of symptoms of depression (with moderate effect size). Previously, mixed results have been found for the effect of interventions on depression. While Morina etal. (15) report a small to medium effect, Purgato etal. (11) did not find any effect. Further, in the present review a beneficial intervention effect was also found for the treatment of PTSD symptoms. Several other studies have reported a beneficial effect of psychological interventions on symptoms of PTSD (11, 15, 43). Moreover, the included interventions had a positive effect on the treatment of anxiety. However, the observed effect was small. In line with our findings, previous studies in low- and middle-income countries have found some- to no beneficial effect for symptoms of anxiety (11, 43). It is not surprising that the effect for anxiety found in this review was small considering the low number of studies examining anxiety. In addition, the interventions included in this review rarely specifically targeted symptoms of anxiety with evidence-based treatment approaches for anxiety such as exposure (44). The only studies which, to our knowledge, aimed to target anxiety with elements of exposure techniques were school-based interventions, however, it remains unclear how exposure was conducted in these group-based interventions. For all outcomes combined and for symptoms of PTSD, the effect size increased after removing outliers. For symptoms of anxiety and depression, the positive effect of treatment decreased when controlling for outliers.

To investigate the association of specific characteristics of the studies with treatment effectiveness, three subgroup analyses were performed in this review. Within the subgroup analyses that were performed, studies utilizing an individual randomization process were compared to studies utilizing cluster randomization. Results show that individual randomized controlled trials showed a higher effect size compared to cluster randomized trials. An explanation may be higher statistical power to detect differences within individual randomized controlled trials. Furthermore, a significant difference in effect size was found between waitlist control condition and non-waitlist control condition, such as treatment-as-usual or no waitlist. Interestingly, interventions of studies implementing non-waitlist condition were found to be more effective than studies utilizing a waitlist condition. This result is not in line with previous studies (10, 45–47) that showed that the effect of psychotherapy is frequently overestimated when treatments are compared to a waitlist control group. Potentially this result may be explained due to the fact that individuals within the waitlist condition were expecting to receive support and therefore experienced a slight relief in symptoms whereas the non-waitlist condition did not experience this and therefore, more pronounced differences between treatment group and control group could be observed. The number of studies included in the subgroup analysis was small, again, also here conclusion must be taken with caution.

Unfortunately, due to the lack of studies with interventions provided in an individual format, no subgroup analysis could be conducted to analyze whether individual therapy is more effective than group therapy. Interestingly, only one study examined an individually delivered intervention, whereas all other studies evaluated interventions for children and adolescents delivered in group format. Given the lack of health care professionals and the high number of individuals in need of treatment, the group format often is considered as a cost-effective and non-invasive solution. Yet, group interventions may also have disadvantages. For example, stigma and shame may become major barriers hindering individuals from sharing private thoughts and experiences related to symptoms of psychological distress. In addition, it may be more challenging to perform evidence-based strategies such as imaginal or *in vivo* exposure in group sessions than in individual sessions (48).

### Limitations

A number of limitations must be noted. Only a total number of thirteen studies was included. Not all of these studies assessed all three outcomes. Hence, when looking at anxiety, depression and symptoms of PTSD separately, the number of studies was even smaller and potentially underpowered. This was also particularly was the case for subgroup analysis. Furthermore, heterogeneity was very high for all outcomes. Studies differed in regard to their methods. Different screening measures were used and time of post-assessments varied. Interventions between studies were very different regarding their session frequency, intensity and content. Therefore, high heterogeneity unfortunately may be inevitable and some issues in aggregating and comparing the data of these different studies must be noted. Moreover, one study (35) included in the analysis reported effect sizes that were exceptionally high, which may suggest that results were overestimated. However, this study was excluded within the sensitivity analysis. Additionally, indications for publication bias were observed, which potentially indicates that the true effect size of psychological treatment may be lower than observed. Lastly, the subgroup analysis on quality of studies did not indicate an association between risk of bias and outcome. Yet, more than half of the studies included were classified as either containing some concerns in regard to risk of bias or containing high risk of bias. As also this subgroup analysis was underpowered, the quality may have still had an effect on outcome. These considerations should be taken into account in light of the results found in this review.

### Clinical Implications

The lack of studies implementing and evaluating psychological and psychosocial interventions for children and adolescents in low- and middle-income countries is striking. Considering, that approximately more than half of the world population lives in low-income and lower-middle-income countries (49) and children and adolescents comprise almost half of the populations in these countries, the number of studies we found on children and adolescents is shockingly low. Evidently, more focus needs to be set upon this population, forging more studies to implement effective interventions in this group. Recommendations for interventions include the cultural adaption of an intervention to its target group before it is implemented (50). In most of the studies included in this review, it was unclear if and how the interventions were culturally adapted. To increase effectiveness and acceptability of interventions, cultural adaption should be carried out. Next to cultural adaption, attention should also be given to caregivers when providing children and adolescents with mental healthcare. To our understanding, in none of the interventions were caregivers included. Yet, studies have shown that caregivers play an important role in increasing or reducing the risk of mental illness in children and adolescents (19, 51). Promising interventions that could be implemented have already been constructed, examples of such interventions are KIDNET and EASE (52–54). ﻿Narrative exposure therapy (NET) has been evaluated in several high quality trials and has been shown to be effective in treating PTSD in adults (55). KIDNET is a narrative exposure therapy for children, which also has been shown to be effective in reducing symptoms of PTSD and increasing levels of functioning (54). EASE was developed for young adolescents and is the adapted version of the World Health Organization (WHO) developed intervention Problem Management Plus (PM+; WHO, 2016). PM+ is a psychological intervention to reduce psychological distress in populations affected by adversities and is delivered by non-professional helpers (56).

### Conclusion

This review suggests that psychological and psychosocial interventions may be effective in reducing symptoms of depression, anxiety, and PTSD for children and adolescents. Results must, however, be considered preliminary since the evidence is not yet strong enough to draw definite conclusions. Due to the large heterogeneity between studies, particularly in regard to the methodological approaches, the combined results of the selected studies must be viewed with caution.

The amount of heterogeneity detected in this meta-analysis certainly limits the conclusions that can be taken from this review. The issues which have contributed to the amount of heterogeneity are the considerably small number of RCTs found that could be included and the variability between the studies found. To allow for a more effective meta-analyses in this field, it is of primary necessity that these issues be addressed. It would be beneficial if research designs and measures are unified across treatment studies (57). Furthermore, researchers should perform complete and transparent reporting on methodological characteristics, and avoid bias that may distort the results (58).

Finally, considering the high prevalence of these common mental health disorders in low- and middle-income countries and the issue of scarce specialized treatment for mental health in these countries, it is essential that psychological treatments be continuously implemented for children and adolescents. Efforts should be made to investigate whether psychological interventions with individual therapy are more effective than group therapies among children and adolescents with high quality trials. Also, when an intervention is to be implemented, considerations should be made as to whether cultural adaption is required first and whether caretakers can and should be included in the intervention.

## Author Contributions

JU led the conduction of the meta-analysis. CA-S was the second rater for full-text ratings, extracted data as a second rater and assessed risk of bias as a second rater. PC advised and supervised the conduction of the meta-analysis. RV was in charge of creating the search string and performing the search in bibliographic databases. MS advised and supervised the conduction of the meta-analysis. All authors were actively involved in writing the article.

## Conflict of Interest

The authors declare that the research was conducted in the absence of any commercial or financial relationships that could be construed as a potential conflict of interest.
